# Analysis of volatile flavor compounds in Antarctic krill paste with different processing methods based on GC‐IMS

**DOI:** 10.1002/fsn3.4425

**Published:** 2024-09-02

**Authors:** Pengfei Jiang, Yang Liu, Jiabo Huang, Baoshang Fu, Kaihua Wang, Zhe Xu

**Affiliations:** ^1^ SKL of Marine Food Processing and Safety Control, National Engineering Research Center of Seafood, School of Food Science and Technology Dalian Polytechnic University Dalian Liaoning China; ^2^ Liaoning Vocational College of Light Industry Dalian Liaoning China; ^3^ College of Life Sciences, Key Laboratory of Biotechnology and Bioresources Utilization Dalian Minzu University, Ministry of Education Dalian Liaoning China

**Keywords:** Antarctic krill paste, electronic nose, flavor optimization, GC‐IMS, processing methods, volatile flavor compounds

## Abstract

In this study, shrimp paste was prepared using Antarctic krill and fermented Antarctic krill shrimp paste as raw materials. Two commonly used heating methods, stir‐fried and steaming, were analyzed, the main difference between the two methods being that stir‐frying involves putting the shrimp paste into a wok and stir‐frying it for different periods of time, while steaming involves putting the shrimp paste into a steamer and steaming it for different periods of time. The effects of different salt concentrations and processing techniques on the volatile flavor compounds of shrimp paste were also observed. Electronic nose and gas chromatography–ion mobility spectrometry (GC‐IMS) were employed to analyze the volatile flavor compounds. A total of 52 volatile flavor compounds were detected by GC‐IMS, of which 38 were identified (including monomers, dimers, and polymers). The identified compounds included 11 aldehydes, 6 ketones, 14 alcohols, 2 esters, 2 acids, 1 pyridine compound, and 2 sulfur compounds. In addition, 14 compounds were identifiable. Using the results of the electronic nose analysis, we were also able to differentiate between the volatile flavor compounds in shrimp pastes produced by different processing methods.

## Graphical abstract

The research aimed to analyze and explore the volatile flavor compounds of eight Antarctic krill pastes using an electronic nose and GC‐IMS. It can be seen that different processing methods have a certain impact on the flavor composition of Antarctic krill pastes.

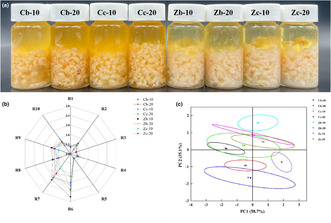

## 1 INTRODUCTION

Shrimp paste is a seasoning made from shrimp through fermentation and is popular in many regions (Amalia et al., 2023; Prihanto et al., 2021). The main shrimp paste in China is in the coastal regions, such as Liaoning, Shandong, Hainan, and Fujian. The raw materials for shrimp paste are diverse, including small shrimp such as silver shrimp, white shrimp, mantis shrimp, and South American white shrimp (Lv et al., 2020; Sang et al., 2020; Yao et al., 2021). Antarctic krill has special high‐protein and low‐fat characteristics, with a protein content of up to 16.31% and a fat content of only 1.3%. Moreover, Antarctic krill is rich in minerals, with a phosphorus content of 2.76%. Its protein hydrolysates also contain a variety of 18 different types of amino acids, including the eight essential amino acids required by the human body. In addition to being rich in nutrients, Antarctic krill also contained various active substances, such as protein digestive enzymes and bacteriocin amino acids (Ding et al., 2023). Antarctic krill paste is, therefore, rich in nutrients (Liu et al., 2018).

In addition, most of the existing studies focus on the fermentation process of shrimp paste, while the effect of shrimp paste processing on the flavor of shrimp paste rarely being reported (Jinap et al., 2010; Surya et al., 2023). The flavor changes of shrimp paste when stir‐fried or steamed with shrimp meat are quite complex (Guzmán‐Guillén et al., 2017; Lu et al., 2022). For flavor stability, different types of flavors were identified using an electronic nose under different cooking time conditions (Hou et al., 2019), and flavor changes were analyzed using GC‐IMS.

Flavor is an important indicator of evaluating the quality of shrimp paste. The electronic nose is an odor detection instrument that can, quickly, accurately, and sensitively distinguish the types of flavor substances contained in the shrimp paste; however, the determination of volatile components is not precise (Fan et al., 2017). The GC‐IMS is a representative instrument that combines a fast separation gas chromatograph (GC) with a highly sensitive ion mobility spectrometer (IMS). It is often used to identify food types and to test the quality and stability of food products (Wang et al., 2020). It is also widely used for the detection of volatile organic compounds in liquids. It is a fairly new technology for volatile component detection that has developed rapidly in recent years, with the following advantages: rapidity, convenience, and visualization. With the increasing use of various combined analytical techniques, it can provide important data to support shelf life information (Jiang et al., 2022; Zhang et al., 2020), variety quality, differentiation of grades (Chen et al., 2021), optimization of processing techniques (Jin et al., 2023; Miao et al., 2023), and origin identification (Jiang et al., 2021). Finally, we combined the two detection methods to analyze flavor changes in the sample.

In this study, fermented Antarctic krill paste mixed with Antarctic krill meat in a proportional ratio was used as the raw material to produce shrimp paste. Different maturation methods were employed, and the differences in the flavor compounds of the shrimp paste were analyzed using an electronic nose and GC‐IMS. The research findings revealed the characteristic flavor compounds of shrimp paste associated with different maturation methods. Different times and salinities have significant effects on different thermal processing modes, and this study provides a reference for the control of flavor quality in the subsequent processing of fermented products, and for the development and evaluation of new products.

## 2 MATERIALS AND METHODS

### 2.1 Preparation of old shrimp paste

Frozen Antarctic krill meat samples were purchased from the Liaoyu Group Co., Ltd (Liaoning, China). Before use, the Antarctic krill meats were thawed in a 4°C cold storage for 12 h. After thawing, the Antarctic krill meat was squeezed with a cheesecloth to remove excess water and placed in a meat grinder (SD‐JR57, Foshan Shunde Sande Electrical Appliance Manufacturing Co., Ltd. China) and ground into a paste. The sample was initially ground at a slow speed for 1 min and then at a fast speed for 1 min, this cycle was repeated twice. Subsequently, the ground paste was removed and mixed evenly with salt (b:20%, c:25%). It was then transferred to a beaker and sealed with a double‐layered cheesecloth. The mixture was fermented at room temperature for 28 days and stirred for 5 min each day.

### 2.2 Preparation of cooked shrimp paste

To prepare cooked shrimp paste, 100 g of old shrimp paste was added at 20% and 100 g at 25% salinity to a mixture of 1000 g of Antarctic krill meat and 500 g of oil and stirred well. For method I, a steaming oven (iCombi Classic, Rational, Germany) was used to steam the samples for 10 or 20 min, and the groups were denoted as ZB‐10, ZB‐20, Zc‐10, and Zc‐20, respectively. For method II, a food processor (Theromix TM6, Vorwerk, Germany) was used to fry the samples for 10 or 20 min, and the groups were denoted as CB‐10, CB‐20, CC‐10, and CC‐20, respectively.

### 2.3 Detection of pathogenic bacteria

*Salmonella*, *Staphylococcus aureus*, *Shigella*, *Listeria monocytogenes*, and *Vibrio parahaemolyticus* were examined according to methods specified in the National Standards of the People's Republic of China.

### 2.4 Measurement of the electronic nose

Volatile flavor compounds in the shrimp paste samples were analyzed using an electronic nose (PEN3, Air Sense Analytics GmbH, Germany). The method by Bai et al. (2020); (Huang et al., 2019) was employed with some modifications. Specifically, 100 mg of each of the eight shrimp paste samples was accurately weighed and incubated at a constant temperature of (60 ± 2)°C in a water bath for 30 min before being analyzed. The electronic nose was rinsed for 20 s and the collection time was 70 s with a sampling interval of 1 s. Three replicates were measured for each sample, and data from 50 to 52 s were selected for analysis. The sensor performances are listed in Table 1.

**TABLE 1:** Sensor sensitivity of electronic nose sensor array.

Sensor number	Sensor name	Performance description
R1	W1C	Sensitive to aromatic components, benzene
R2	W5S	High sensitivity, sensitive to aromatic components
R3	W3C	Sensitive to ammonia, aromatic components
R4	W6S	Selective for hydrides
R5	W5C	Short‐chain alkanes, aromatic components
R6	W1S	Sensitive to methyl groups
R7	W1W	Sensitive to inorganic sulfides
R8	W2S	Sensitive to alcohols, aldehydes, ketones
R9	W2W	Sensitive to organic sulfides
R10	W3S	Sensitive to long‐chain alkanes

### 2.5 Measurement of gas chromatography–ion mobility spectrometry

The shrimp paste sample (1.0 g) was loaded into a headspace vial (20.0 mL) in keeping with the method described by Jiang et al. (2024) with slight modifications. The injection conditions were as follows: incubation temperature of 60°C, incubation time of 15 min, injection volume of 500 μL, splitless injection, incubation speed of 500 r/min, and an injection needle temperature of 85°C. The GC conditions were as follows: the column temperature was set at 60°C, high‐purity nitrogen (purity ≥99.999%) was used as the carrier gas, and the programmed boosting was as follows: from an initial flow rate of 2.0 mL/min for 2 min, the flow rate increased linearly to 10.0 mL/min within 8 min, then to 100.0 mL/min within 10 min, and to 150.0 mL/min within the last 10 min. The total running time of the chromatography was 30 min, and the temperature of the injection port was 80°C. The ionization source was selected as tritium source (3H), the migration tube length was 53 mm, the electric field strength was 500 V/cm, the migration tube temperature was 45°C, the drift gas was selected as high‐purity nitrogen (purity ≥99.999%), the flow rate was controlled at 150.0 mL/min, and the positive ion mode was selected.

### 2.6 Sensory evaluation

Sensory evaluation was done with appropriate modifications following the methodology of Li et al. (2021). Ten trained team members, five boys and five girls, were selected to evaluate the organoleptic characteristics of shrimp paste samples under different processing methods from five perspectives: color, form, flavor, taste, and overall acceptability. The researchers had researchers rate each trait on a scale of 0–10, with 0 being the smallest and 10 being the largest. The average sensory score for each trait for each group of thermally processed shrimp paste was calculated by averaging the scores of all group members.

### 2.7 Statistical analysis

The target compounds were qualitatively analyzed using a GC‐IMS system with VOCal software, which incorporates the GC retention index (NIST 2020) database and IMS migration time database for retrieval and alignment. The Reporter, Gallery Plot, and Dynamic PCA plugins in the VOCal data processing software were utilized to generate three‐dimensional spectra, two‐dimensional spectra, difference spectra, fingerprint spectra, and PCA plots of volatile components, enabling the comparison of volatile organic compounds among the samples. WinMuster software was used for the electronic nose detection analysis. All data were analyzed for significance using Duncan's test and one‐way ANOVA in SPSS (version 20.0) and plotted using Origin 2021.

## 3 RESULTS AND DISCUSSION

### 3.1 Results of pathogenic bacteria

To ensure the safety of the experiment, the pathogenic bacteria in the 28‐day fermented Antarctic shrimp paste were tested before cooking. The test results showed that no pathogens were detected, thus meeting the hygiene standards and ensuring food safety (see Table 2 for detailed results).

**TABLE 2:** Levels of pathogenic bacteria in old shrimp paste fermented for 28 days.

Testing programs	Unit	Detection methods	Test results	
			20% (b)	25% (c)
*Salmonella*	/25 g	GB 4789.4–2016	ND	ND
*Staphylococcus aureus*	/25 g	GB 4789.10–2016 the first method	ND	ND
*Shigella*	/25 g	GB 4789.5–2012	ND	ND
*Listeria monocytogenes*	CFU/g	GB 4789.30–2016 the second method	<10	<10
*Vibrio parahaemolyticus*	MPN/g	GB 4789.7–2013 5.2.2	<0.3	<0.3

### 3.2 Results of electronic nose

**FIGURE 1:**
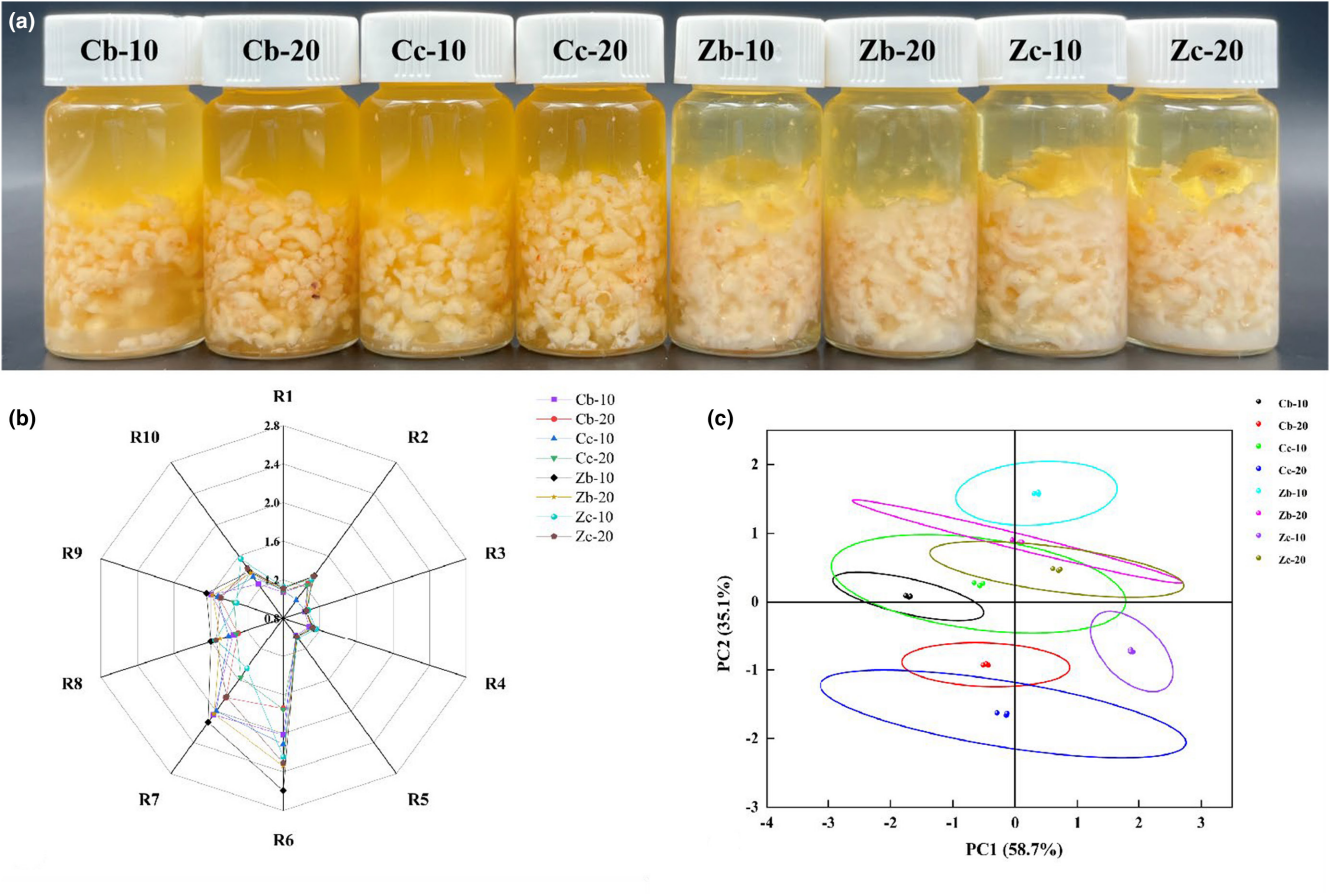
Shrimp paste samples fried and steamed (a) with different salt concentrations after oil addition; Radar plots (b) and PCA analysis (c) of sensor responses for different shrimp paste sample odors.

A certain amount of Antarctic krill mince (Figure 1a) was steamed and fried at different times with different salt concentrations in shrimp paste. It can be seen that there is a difference in meat quality between stir frying and steaming. The color of the oil during steaming was light yellow, whereas that during stir‐frying was golden yellow. The radar chart in Figure 1b shows the sensor responses to different Antarctic krill paste sample odors under different processing techniques. It can be seen from the chart that the differences among the eight volatile flavor substances in the Antarctic krill paste were mainly concentrated in sensors R6, R7, R8, R9, and R10, with higher response values. The Zb‐10 sample had the highest response values in sensors R6, R7, R8, and R9, whereas all the samples showed significant differences in sensors R6 and R7. This indicates significant differences in methyl and inorganic sulfides among the eight shrimp paste samples. The shrimp pastes with higher response values for sensors R6 and R7 were those from the steaming group. Lu et al. (2022) detected methyl compounds as key flavor compounds in steamed shrimp paste, whereas Zhu et al. (2019) found that electronic nose sensors showed higher responses to inorganic sulfides. The responses of sensors R1, R3, R4, and R5 were relatively similar, indicating that the volatile flavor substances represented by these sensors in the eight samples were similar.

Based on the PCA analysis of different Antarctic krill samples using electronic nose response values, as shown in Figure 1c, the contribution rates of PC1 and PC2 were 58.7% and 35.1%, respectively, and the cumulative contribution rate reached 93.8% (greater than 90%), which included the main characteristics of the volatile flavor compounds in the eight shrimp paste samples. The steamed samples were mainly concentrated on the positive half‐axis of PC1, whereas the stir‐fried samples were concentrated on the negative half‐axis of PC1. The Zb‐10 sample did not overlap with other samples and could be well distinguished; there was partial overlap between Zc‐10 and Cc‐10, as well as between Cb‐20 and Cc‐20, indicating some similarity in volatile flavor compounds among these groups of samples; there was overlap among Zb‐20, Zc‐20, Cc‐10, and Cb‐10, indicating a high degree of similarity in volatile flavor compounds among these groups of samples.

### 3.3 Volatile compounds identified in shrimp paste by GC‐IMS

#### 3.3.1 Analysis of flavor composition spectrum

**FIGURE 2:**
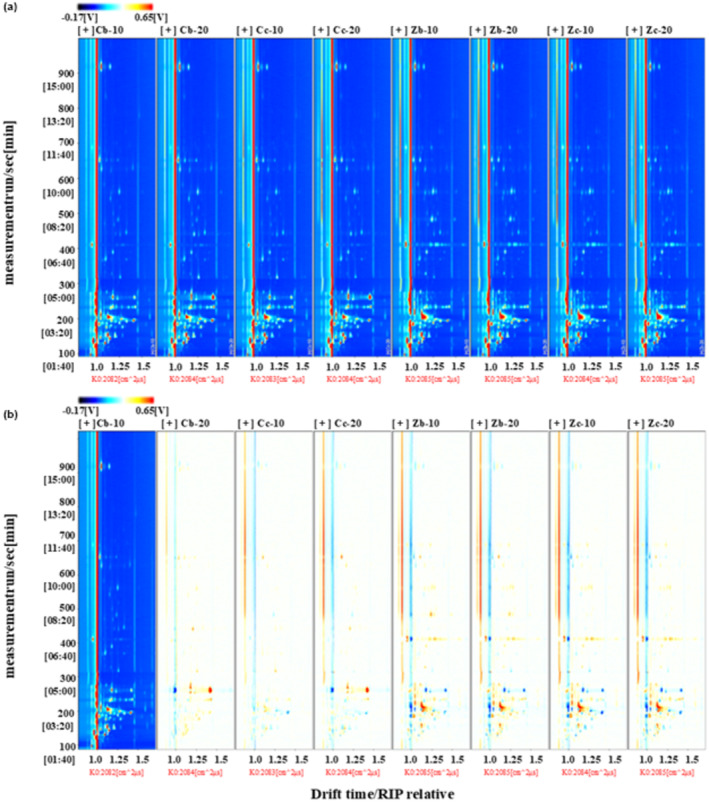
Two‐dimensional (a) and differential spectra (b) of volatile organic compounds (VOCs) in different shrimp paste samples by GC‐IMS.

The two‐dimensional spectra of volatile flavors in the samples of the eight Antarctic krill instant shrimp pastes are shown in Figure 2a. The vertical axis represents the GC retention time in seconds, and the horizontal axis represents the relative movement time. Each point represents a VOC on either side of the RIP peak. The color represents the peak intensity of the substance; the darker the color, the stronger the peak. The composition of the volatile compounds in the different shrimp pastes varied. The ion migration time of volatile compounds in Antarctic krill paste by different production methods is mainly concentrated between 1.0 and 1.5 ms, and the retention time is concentrated between 150 and 450 s.

#### 3.3.2 GC‐IMS fingerprint analysis

**FIGURE 3:**
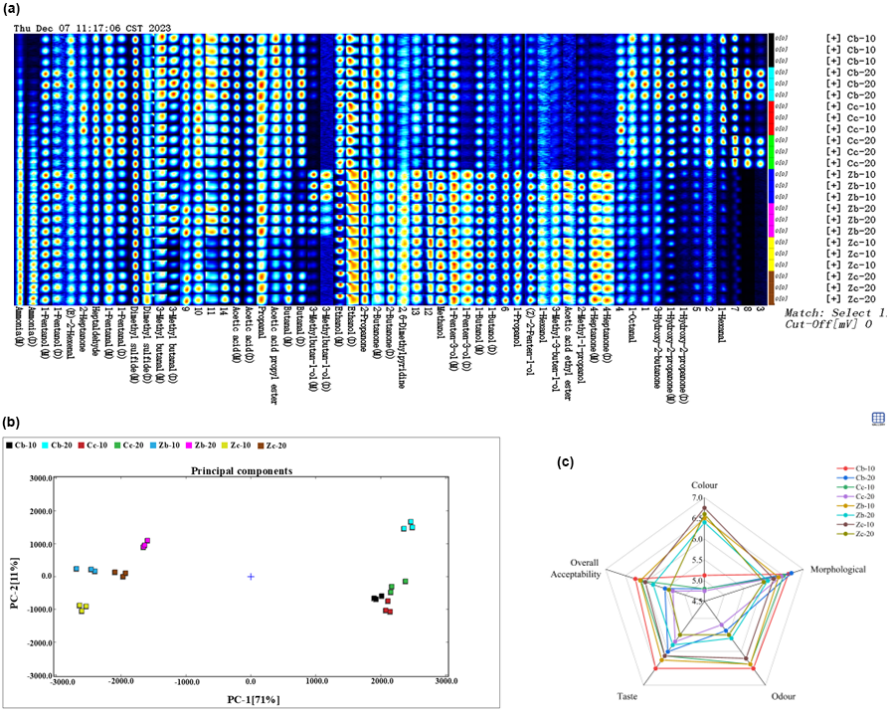
Fingerprinting of volatiles in different shrimp paste samples (a), principal component analysis (b), and sensory evaluation (c).

To further analyze the changes in the volatile flavor compounds of Antarctic krill sauce, a qualitative analysis of the detected substances was conducted using the built‐in database of the software based on the migration and retention times (Li et al., 2022). The fingerprint chromatograms of the eight volatile flavor compounds in Antarctic krill sauce are shown in Figure 3, where each row represents all the volatile flavor compounds in a sample, and each column represents the content of the same volatile flavor compound in different Antarctic krill paste samples. The darker the red color, the higher the substance content, whereas the darker the blue color, the lower the substance content. “M” represents monomers, and “D” represents dimers. A total of 52 volatile flavor compounds were detected by GC‐IMS, of which 38 were identified qualitatively (including monomers, dimers, and polymers). There were 11 aldehydes, 6 ketones, 14 alcohols, 2 esters, 2 acids, 1 pyridine, and 2 sulfur‐containing compounds. Fourteen compounds were unidentifiable.

As shown in Figure 3, there were significant differences in the volatile compounds between the different production methods for Antarctic krill paste, particularly between the stir‐fried and steamed groups. The substances highlighted in the red box in Figure 3 had a higher content in the stir‐fried Antarctic krill paste, whereas they were almost absent in the steamed Antarctic krill. These substances include hexanol, 1‐hydroxy‐2‐propanone, 3‐hydroxy‐2‐butanone, and 1‐octanal. The substances highlighted in the yellow box contained higher concentrations of steamed shrimp paste, including 4‐heptanone, 2‐methyl‐1‐propanol, acetic acid ethyl ester, 3‐methyl‐3‐buten‐1‐ol, 1‐hexanol, (Z)‐2‐pentenal‐1‐ol, 1‐propanol, 1‐butanol, 1‐penten‐3‐ol, methanol, 2,6‐dimethylpyridine, 2‐butanone, 2‐propanone, and ethanol.

#### 3.3.3 Relative content analysis

The composition and percentage of volatile flavor substances identified in the eight Antarctic krill sauces are shown in Table 3 and Table S1. Aldehydes have a lower threshold and a higher contribution to the main flavor of shrimp sauces. There are two main ways to form aldehydes, one by fatty acid oxidation during the fermentation process, and the other by the Maillard reaction (Lu et al., 2022). Aldehydes with 6–10 carbon atoms are the main volatile compounds produced by lipid oxidation, including hexanal, butanal, propanal, octanal, nonanal, and heptanal (Li et al., 2024). The 11 aldehydes detected in relatively high contents were: 1‐octanal, (E)‐2‐hexenal, heptaldehyde, 1‐hexanal, 1‐pentanal monomer and dimer, butanal monomer and dimer, 3‐methyl butanal monomer and dimer, and propanal. Among them, the propanal found to have the highest content had a cocoa‐like, earthy, and ethereal aroma. 1‐pentanal, 1‐hexanal, heptaldehyde, 1‐octanal, etc. from the autooxidation and enzymatic oxidation of unsaturated fatty acids are mainly composed of aliphatic aldehydes (Purriños et al., 2013). 1‐hexanal presented aromas similar to grass, tallow, and fat (Li, Zhang, et al., 2023). 1‐pentanal was mainly produced by the oxidation and decomposition of fatty acids (Domínguez et al., 2019), and among them, 1‐pentanal had an almond‐like, malty, and pungent aroma (Li, Zhang, et al., 2023). 3‐methyl butanal exhibited fruity and phosphate‐like flavors. It was detected in shrimp paste samples, as reported by (Luo, 2022). The total content of aldehydes was higher in the fried group than in the steamed group, which could be attributed to the absorption of unsaturated fatty acids from the oil during frying, leading to increased lipid oxidation in the fried group. Frying also accelerated this process, leading to increased aldehyde content in the fried group (Luo et al., 2022). Cb‐10 was higher than Cb‐20 in the stir‐fry group due to partial loss of aldehydes as a result of prolonged stir‐frying time.

**TABLE 3:** Key volatile compounds in different shrimp paste samples detected by GC‐IMS.

Compound class	Key compounds	CAS#	Formula	MW	RI	Rt [sec]	Dt [a.u.]	Odor description
Aldehydes (11)	1‐Octanal	C124130	C_8_H_16_O	128.2	1294.7	628.973	1.40803	Fat, soap, lemon, green
	(E)‐2‐Hexenal	C6728263	C_6_H_10_O	98.1	1225.7	499.437	1.18858	Green, leaf, apple
	Heptaldehyde	C111717	C_7_H_14_O	114.2	1190.3	443.604	1.33186	Fat, citrus, rancid
	1‐Hexanal	C66251	C_6_H_12_O	100.2	1096.8	317.891	1.2667	Grass, tallow, fat
	1‐Pentanal(M)	C110623	C_5_H_10_O	86.1	992	233.435	1.18245	Almond, malt, pungent
	1‐Pentanal(D)	C110623	C_5_H_10_O	86.1	989.6	231.983	1.42894	Almond, malt, pungent
	Butanal(M)	C123728	C_4_H_8_O	72.1	879.1	175.326	1.11158	Pungent, green
	Butanal(D)	C123728	C_4_H_8_O	72.1	881.1	176.231	1.2932	Pungent, green
	3‐Methyl butanal(M)	C590863	C_5_H_10_O	86.1	919.4	194.216	1.16586	Malt
	3‐Methyl butanal(D)	C590863	C_5_H_10_O	86.1	919.9	194.451	1.41503	Malt
	Propanal	C123386	C_3_H_6_O	58.1	802.1	144.255	1.14981	Cocoa, earthy, ethereal
Ketones (9)	2‐Heptanone	C110430	C_7_H_14_O	114.2	1188.6	440.888	1.26346	Soap
	2‐Propanone	C67641	C_3_H_6_O	58.1	824.3	152.618	1.12378	Minty chemical, sweet, solventy
	2‐Butanone(M)	C78933	C_4_H_8_O	72.1	904.9	187.185	1.06292	Fragrant, fruit, pleasant
	2‐Butanone(D)	C78933	C_4_H_8_O	72.1	905.9	187.653	1.25762	Fragrant, fruit, pleasant
	4‐Heptanone(M)	C123193	C_7_H_14_O	114.2	1168.7	410.714	1.22207	
	4‐Heptanone(D)	C123193	C_7_H_14_O	114.2	1168	409.714	1.58586	
	1‐Hydroxy‐2‐propanone(M)	C116096	C_3_H_6_O_2_	74.1	1312.7	652.903	1.03138	Tobacco‐like
	1‐Hydroxy‐2‐propanone(D)	C116096	C_3_H_6_O_2_	74.1	1311.2	650.909	1.24101	Tobacco‐like
	3‐Hydroxy‐2‐butanone	C513860	C_4_H_8_O_2_	88.1	1292.8	624.985	1.05605	Butter, cream
Alcohols (16)	1‐Pentanol(M)	C71410	C_5_H_12_O	88.1	1260.3	560.662	1.25296	Radishes, vegetables
	1‐Pentanol(D)	C71410	C_5_H_12_O	88.1	1259.6	559.458	1.51406	Radishes, vegetable
	3‐Methylbutan‐1‐ol(M)	C123513	C_5_H_12_O	88.1	1215.5	482.778	1.24763	Whiskey, malt, burnt
	3‐Methylbutan‐1‐ol(D)	C123513	C_5_H_12_O	88.1	1215.7	483.132	1.49567	Whiskey, malt, burnt
	1‐Butanol(M)	C71363	C_4_H_10_O	74.1	1154.1	389.845	1.18139	Medicine, fruit
	1‐Butanol(D)	C71363	C_4_H_10_O	74.1	1154.3	390.229	1.38175	Medicine, fruit
	2‐Methyl‐1‐propanol	C78831	C_4_H_10_O	74.1	1106.5	328.996	1.17023	Alcohol, fruity, banana
	1‐Propanol	C71238	C_3_H_8_O	60.1	1049.8	276.523	1.11624	Alcohol, pungent
	Methanol	C67561	CH_4_O	32	899.8	184.779	1.0285	
	Ethanol(M)	C64175	C_2_H_6_O	46.1	939.2	204.191	1.04044	Sweet
	Ethanol(D)	C64175	C_2_H_6_O	46.1	937.2	203.173	1.13151	Sweet
	1‐Penten‐3‐ol(M)	C616251	C_5_H_10_O	86.1	1169.8	412.431	0.94852	Wine, leather
	1‐Penten‐3‐ol(D)	C616251	C_5_H_10_O	86.1	1169	411.119	1.34566	Wine, leather
	3‐Methyl‐3‐buten‐1‐ol	C763326	C_5_H_10_O	86.1	1260.2	560.413	1.16519	
	(Z)‐2‐Penten‐1‐ol	C1576950	C_5_H_10_O	86.1	1340.2	690.912	0.9492	Green, plastic, rubber
	1‐Hexanol	C111273	C_6_H_14_O	102.2	1370.4	735.208	1.32517	Resin, flower, green
Esters (2)	Acetic acid ethyl ester	C141786	C_4_H_8_O_2_	88.1	888.6	179.619	1.3433	Potatoes, roasted nuts
	Acetic acid propyl ester	C109604	C_5_H_10_O_2_	102.1	985.4	229.577	1.1577	Potatoes, roasted nuts
Acids (2)	Acetic acid(M)	C64197	C_2_H_4_O_2_	60.1	1447.7	919.304	1.05421	Sour, vinegar
	Acetic acid(D)	C64197	C_2_H_4_O_2_	60.1	1448.4	920.871	1.15009	Sour, vinegar
Ethers (2)	Dimethyl sulfide(M)	C75183	C_2_H_6_S	62.1	774.5	134.544	0.9623	
	Dimethyl sulfide(D)	C75183	C_2_H_6_S	62.1	776.9	135.359	1.12893	
Pyridines	2,6‐Dimethylpyridine	C108485	C_7_H_9_N	107.2	1272.9	584.865	1.08587	
Unidentified (14)	1	*	*	0	1250.8	543.196	1.15785	
	2	*	*	0	1236.5	517.9	1.26508	
	3	*	*	0	1189.2	441.876	1.21524	
	4	*	*	0	1140.7	371.748	1.10162	
	5	*	*	0	1138.2	368.416	1.33423	
	6	*	*	0	1127	353.981	1.0909	
	7	*	*	0	1030.6	261.273	1.18245	
	8	*	*	0	1029	260.063	1.42097	
	9	*	*	0	890.1	180.303	1.16413	
	10	*	*	0	811	147.578	1.04432	
	11	*	*	0	813.2	148.392	1.2187	
	12	*	*	0	697.6	110.718	1.06757	
	13	*	*	0	727	119.271	1.13086	
	14	*	*	0	759.6	129.526	1.09499	

Among these, oxidative degradation of unsaturated fatty acids, degradation of amino acids, and microbial activity are important sources of ketones in shrimp paste. They possess floral and fruity aromas, and were also the reason for the formation of a cheese‐like aroma in fish sauce (Rossana et al., 1996).

Among the nine detected ketone substances, the one found in the highest concentration was 2‐propanone, which has a slightly chemical‐like but sweet taste (Li, Zhang, et al., 2023). The steamed shrimp paste had a higher content of 2‐propanone compared to the stir‐fried shrimp paste. Carbohydrates undergo microbial fermentation to produce various volatile flavor compounds. Shrimp paste involves a wide range of species in the fermentation process (Huang et al., 2024). However, some microorganisms in shrimp paste use carbohydrates as a carbon source for their own growth and reproduction and produce organic acids such as pyruvic acid through the glycolytic pathway (Qin et al., 2024), which is the main source of acidity in fermented products.

Alcohol compounds had lower contributions to the flavor of the Antarctic krill paste because of their high flavor threshold. Alcohols are usually derived from the oxidation and degradation of fats as well as the cleavage of fatty acid carbon chains. The resulting free radicals can combine with hydroxyl radicals to form alcohols, which can impart a pleasant aroma to aquatic products (Fu et al., 2022). Although alcohol compounds were not the most abundant in terms of variety, they had a high relative content. This result is consistent with Zhao et al. (2019).

Two lipids were also detected: acetic acid ethyl ester and acetic acid propyl ester. Acetic acid ethyl ester has a fragrance similar to potatoes and roasted nuts (Li, Yuan, et al., 2023). Esters are generally produced by esterification reactions or lipid oxidation and have fruity and sweet aromas (Yang et al., 2023). Their flavor can enhance the taste of aquatic products; however, owing to its high threshold, it does not contribute significantly to the flavor. Acetic acid may have been produced through fermentation of insoluble calcium in the shrimp shells, leading to the formation of a large amount of soluble calcium, beneficial for human consumption (Li, Leng, et al., 2023). The Cb‐10 content was higher in the fried group followed by Cb‐20 and less in the other groups especially in the steamed group.

Microorganisms in fermented aquatic products not only produce various aromatic substances during their action but also generate unpleasant odor substances, such as dimethyl sulfide (Li, Mi, Liu, Sang, & Sun, 2022). Dimethyl sulfide (M) is relatively more abundant in Cb‐10, while dimethyl sulfide (D) is relatively more abundant in Cb‐20 and less abundant in the other groups. But Ding et al. (2013) detected that dimethyl sulfide has a relatively high content in the meat of Antarctic shrimp, and is a key flavor component of shrimp meat.

From the relative content of the flavor compounds, it can be seen that the flavor compounds were more abundant in the Cb‐10 group followed by Cb‐20 in the stir‐frying group and Zb‐10 in the steaming group, the reason being that the loss of flavor compounds was more serious with the stir‐frying and steaming time; however, the relative content of the flavor compounds in Cc‐10, Cc‐20, Zc‐10, and Zc‐20 was less. However, Cc‐10, Zc‐10, and Zc‐20 flavor compounds were relatively less due to the high salinity which resulted in low content of flavor compounds.

Figure 3b shows the principal component analysis (PCA) score plot of the stir‐fried and steamed shrimp pastes. The contribution of the two principal components was 11% and 71%, respectively, with a cumulative contribution rate of 82%. This indicates that the principal components effectively reflect the overall information of the samples and can be used for flavor data analysis. The plot visually displays the differences between different samples, where smaller distances between samples represent smaller differences and wider distances represent more significant differences. It can be observed that there are significant differences in volatile organic compounds between stir‐fried and steamed shrimp paste. The four groups of samples in the stir‐fried group were concentrated on the left side, while the samples in the steamed group were concentrated on the right. Each sample on both sides could be separated, indicating good differentiation of volatile flavors among the groups of samples.

#### 3.3.4 Sensory evaluation analysis

Sensory evaluation of eight groups of shrimp paste in two heating methods, frying and steaming, was carried out on color, form, odor, taste, and overall acceptability. As shown in Figure 3c, the morphological changes in shrimp paste after thermal processing were not significant in the eight groups of samples. The highest flavor, taste, and overall acceptability flavor were found in the Cb‐10 group, followed by higher shrimp paste made for 10 min in the steaming and stir‐frying groups, respectively, which is consistent with the high volatile compounds content in the GC‐IMS due to the loss of most of the volatile compounds from the prolonged stir‐frying and steaming. There was a significant difference in color between the steamed and stir‐fried groups in the sensory because the shrimp paste in the steamed group was made more translucent by the action of water vapor in the steamer, whereas in the stir‐fried group after repeated stir‐frying the higher the salinity and the longer the color was heavier making it a lower score. It was found that the flavor profile of the low salinity heating method with short time was more preferred. The results suggest that the processing method, time, and salinity are the main factors affecting the quality of shrimp paste.

## 4 CONCLUSIONS

This study aimed to analyze and explore the volatile flavor compounds in eight Antarctic krill pastes using an electronic nose and GC‐IMS technology. Substances such as 1‐hexanol, 1‐hydroxy‐2‐propanone, 3‐hydroxy‐2‐butanone, and 1‐octanal were present in high levels in the stir‐fried group but were almost absent in the steamed group. In contrast, substances such as 4‐heptanone, 2‐methyl‐1‐propanol, acetic acid ethyl ester, 3‐methyl‐3‐buten‐1‐ol, 1‐hexanol, (E)‐2‐hexenal, propanal, 1‐butanol, 1‐penten‐3‐ol, methanol, 2,6‐dimethylpyridine, 2‐butanone, 2‐propanone, and ethanol were present in high levels in the steamed group. There were significant differences in the volatile compounds of the Antarctic krill pastes processed using different methods. Combined with the results of the electronic nose analysis, it can be concluded that R6 and R7 are characteristic sensors. The flavor substances of the Zb‐10 sample were well separated from the other components, while there was some overlap of volatile flavor compounds in the other groups of samples. Therefore, it can be seen that different processing methods have a certain impact on the flavor composition of Antarctic krill pastes.

## AUTHOR CONTRIBUTIONS

**Pengfei Jiang:** Formal analysis (equal); funding acquisition (equal); project administration (equal); writing – original draft (lead). **Yang Liu:** Data curation (lead); methodology (lead). **Jiabo Huang:** Software (lead). **Baoshang Fu:** Formal analysis (equal); methodology (equal). **Kaihua Wang:** Writing – review and editing (equal). **Zhe Xu:** Writing – original draft (equal); writing – review and editing (equal).

## CONFLICT OF INTEREST STATEMENT

All authors declare that they have no conflicts of interest.

## Supporting information


Table S1.

## Data Availability

Data are available on request from the authors.
