# Supplementary material for: Analysis of volatile flavor compounds in Antarctic krill paste with different processing methods based on GC‐IMS
Source: Food Sci Nutr. 2024 Sep 2;12(10):8353–63. doi: 10.1002/fsn3.4425 (PMC11521673; doi:10.1002/fsn3.4425)
Supplement: Supplementary file 1 — Table S1. [file FSN3-12-8353-s001.docx]

**Table 3S** Relative content of key volatile compounds in different shrimp paste samples detected by GC-IMS.

| Compound class | Key  compounds | Relative content% | | | | | | | |
| --- | --- | --- | --- | --- | --- | --- | --- | --- | --- |
|  |  | Cb10 | Cb20 | Cc10 | Cc20 | Zb10 | Zb20 | Zc10 | Zc20 |
| Aldehydes（11） | 1-Octanal | 0.35±0.03^a^ | 0.29±0.05^b^ | 0.21±0.03^c^ | 0.19±0.02^c^ | 0.11±0.01^d^ | 0.1±0.01^d^ | 0.11±0.01^d^ | 0.1±0^d^ |
|  | (E)-2-Hexenal | 0.34±0.02^a^ | 0.24±0.01^b^ | 0.18±0.01^c^ | 0.15±0.02^de^ | 0.16±0.02^cd^ | 0.13±0.01^e^ | 0.13±0^e^ | 0.13±0^e^ |
|  | Heptaldehyde | 0.62±0.07^a^ | 0.59±0.06^ab^ | 0.52±0.02^b^ | 0.41±0.06^c^ | 0.34±0^cd^ | 0.34±0.02^cd^ | 0.3±0.02^d^ | 0.31±0.01^d^ |
|  | 1-Hexanal | 0.91±0.32^a^ | 0.82±0.14^a^ | 0.87±0.06^a^ | 0.51±0.15^b^ | 0.23±0.07^c^ | 0.2±0.03^c^ | 0.15±0.02^c^ | 0.19±0.05^c^ |
|  | Pentanal(M) | 1.52±0.09^a^ | 1.35±0.08^b^ | 1.2±0.05^c^ | 1.1±0.08^c^ | 0.72±0.01^cd^ | 0.81±0.02^c^ | 0.68±0.02^d^ | 0.8±0.04^c^ |
|  | 1-Pentanal(D) | 1.11±0.13^a^ | 1.44±0.15^b^ | 1.1±0.05^b^ | 1.04±0.12^b^ | 0.61±0.02^de^ | 0.82±0.03^c^ | 0.48±0.04^e^ | 0.71±0.08^cd^ |
|  | Butanal(M) | 1.48±0.06^a^ | 1.25±0.09^b^ | 1.13±0.04^c^ | 0.9±0.06^d^ | 0.57±0.01^f^ | 0.76±0.01^e^ | 0.5±0.01^f^ | 0.74±0^e^ |
|  | Butanal(D) | 0.78±0.04^a^ | 0.91±0.05^b^ | 0.66±0.04^c^ | 0.56±0.03^d^ | 0.19±0.01^f^ | 0.44±0.03^e^ | 0.15±0.01^f^ | 0.4±0.01^e^ |
|  | 3-Methylbutanal(M) | 1.39±0.06^a^ | 0.99±0.09^b^ | 0.85±0.05^c^ | 0.73±0.03^d^ | 0.58±0.03^e^ | 0.56±0.02^e^ | 0.52±0.01^e^ | 0.53±0.01^e^ |
|  | 3-Methyl butanal(D) | 2.91±0.31^b^ | 2.56±0.16^c^ | 1.16±0.03^e^ | 1.18±0.09^e^ | 1.09±0.02^e^ | 1.48±0.03^d^ | 0.47±0.02^f^ | 0.73±0^f^ |
|  | Propanal | 2.56±0.06^a^ | 2.22±0.18^b^ | 2.03±0.08^c^ | 1.62±0.04^d^ | 1.29±0.02^e^ | 1.6±0.01^d^ | 1.15±0.02^e^ | 1.55±0.02^d^ |
| Total |  | 13.96±1.14^a^ | 12.65±1.06^b^ | 9.9±0.42^c^ | 8.39±0.68^d^ | 5.89±0.2^fg^ | 7.23±0.08^de^ | 4.64±0.12^g^ | 6.17±0.14^ef^ |
| Ketones（9） | 2-Heptanone | 0.33±0.02^a^ | 0.27±0.02^b^ | 0.36±0.01^a^ | 0.22±0.01^c^ | 0.21±0.01^cd^ | 0.19±0.01^d^ | 0.21±0.02^cd^ | 0.19±0^d^ |
|  | 2-Propanone | 6±0.11^a^ | 5.36±0.41^b^ | 3.99±0.21^e^ | 2.92±0.03^f^ | 5.02±0.1^bc^ | 4.69±0.1^cd^ | 4.61±0.06^d^ | 4.53±0.06^d^ |
|  | 2-Butanone(M) | 0.5±0.01^a^ | 0.4±0.02^b^ | 0.35±0.02^c^ | 0.28±0.01^d^ | 0.25±0.01^e^ | 0.25±0^e^ | 0.26±0^de^ | 0.25±0.01^e^ |
|  | 2-Butanone(D) | 0.87±0.05^ab^ | 0.91±0.04^a^ | 0.53±0.04^d^ | 0.43±0.01^e^ | 0.92±0.02^a^ | 0.91±0.01^a^ | 0.76±0.01^c^ | 0.83±0.03^b^ |
|  | 4-Heptanone(M) | 0.16±0^c^ | 0.14±0.02^c^ | 0.11±0.01^d^ | 0.11±0.01^d^ | 0.37±0.02^a^ | 0.33±0.01^b^ | 0.39±0.01^a^ | 0.38±0.01^a^ |
|  | 4-Heptanone(D) | 0.06±0.01^d^ | 0.05±0^e^ | 0.04±0.01^e^ | 0.04±0^e^ | 0.13±0.01^a^ | 0.1±0.01^c^ | 0.11±0.01^b^ | 0.12±0^ab^ |
|  | 1-Hydroxy-2-propanone(M) | 1.8±0.56^ab^ | 1.93±0.07^a^ | 1.39±0.22^bc^ | 1.15±0.05^c^ | 0.67±0.15^d^ | 0.65±0.04^d^ | 0.47±0.07^d^ | 0.43±0.03^d^ |
|  | 1-Hydroxy-2-propanone(D) | 0.25±0.11^b^ | 0.34±0.02^a^ | 0.23±0.05^b^ | 0.2±0.01^b^ | 0.08±0.02^d^ | 0.09±0.01^d^ | 0.06±0.01^d^ | 0.07±0.01^d^ |
|  | 3-Hydroxy-2-butanone | 1.21±0.15^a^ | 1.06±0.05^b^ | 0.81±0.1^c^ | 0.62±0.03^d^ | 0.45±0.03^e^ | 0.45±0.01^e^ | 0.39±0.02^e^ | 0.39±0.01^e^ |
| Total |  | 11.19±0.73^a^ | 10.46±0.62^a^ | 7.81±0.66^bc^ | 5.98±0.04^d^ | 8.11±0.31^b^ | 7.64±0.06^bc^ | 7.26±0.12^bc^ | 7.2±0.07^c^ |
| Alcohols（16） | 1-Pentanol(M) | 0.98±0^b^ | 1.07±0.08^a^ | 0.75±0.07^d^ | 0.65±0.05^e^ | 0.85±0.01^c^ | 0.8±0.01^cd^ | 0.81±0^cd^ | 0.73±0.03^de^ |
|  | 1-Pentanol(D) | 0.15±0.02^a^ | 0.15±0.02^a^ | 0.11±0.03^b^ | 0.09±0.02^b^ | 0.12±0.01^ab^ | 0.11±0.02^b^ | 0.11±0.01^b^ | 0.11±0.01^b^ |
|  | 3-Methylbutan-1-ol(M) | 0.49±0.01^c^ | 0.36±0.01^d^ | 0.16±0.03^g^ | 0.12±0.01^g^ | 1.01±0.03^a^ | 0.73±0.04^b^ | 0.27±0.02^e^ | 0.22±0^f^ |
|  | 3-Methylbutan-1-ol(D) | 0.08±0.01^b^ | 0.06±0^c^ | 0.05±0.02^c^ | 0.04±0^d^ | 0.15±0^a^ | 0.09±0.01^b^ | 0.03±0^d^ | 0.03±0^d^ |
|  | 1-Butanol(M) | 0.74±0.03^a^ | 0.63±0.05^cd^ | 0.53±0.02^e^ | 0.47±0.01^f^ | 0.68±0.01^b^ | 0.59±0^d^ | 0.64±0.01^bc^ | 0.59±0.01^d^ |
|  | 1-Butanol(D) | 0.09±0.01^d^ | 0.08±0.01^de^ | 0.06±0.02^ef^ | 0.04±0^f^ | 0.21±0.01^a^ | 0.17±0.01^c^ | 0.2±0.01^ab^ | 0.19±0^bc^ |
|  | 2-Methyl-1-propanol | 0.2±0.02^bc^ | 0.18±0.03^c^ | 0.14±0.02^d^ | 0.12±0.02^d^ | 0.29±0.02^a^ | 0.22±0.01^b^ | 0.24±0.01^b^ | 0.22±0.01^bc^ |
|  | 1-Propanol | 0.43±0.01^c^ | 0.36±0.03^d^ | 0.3±0.02^e^ | 0.24±0.01^f^ | 0.53±0.02^a^ | 0.5±0.01^ab^ | 0.49±0.01^b^ | 0.5±0.01^b^ |
|  | Methanol | 2.28±0.04^c^ | 1.47±0.07^e^ | 1.52±0.03^e^ | 1.35±0.03^f^ | 2.42±0.05^b^ | 2.15±0.07^d^ | 2.52±0.02^a^ | 2.21±0.03^cd^ |
|  | Ethanol(M) | 4.53±0.11^a^ | 3.33±0.22^b^ | 3.3±0.14^b^ | 2.77±0.08^c^ | 1.98±0.06^d^ | 1.99±0.03^d^ | 2.02±0.01^d^ | 2.02±0.01^d^ |
|  | Ethanol(D) | 5.87±0.11^a^ | 4.24±0.31^c^ | 4.91±0.26^b^ | 3.52±0.19^e^ | 4.01±0.04^cd^ | 3.97±0.06^cd^ | 3.87±0.05^d^ | 3.7±0.05^de^ |
|  | 1-Penten-3-ol(M) | 2.22±0.06^c^ | 1.81±0.07^d^ | 1.4±0.05^e^ | 1.22±0.02^f^ | 2.38±0.03^a^ | 2.25±0.01^bc^ | 2.32±0.01^ab^ | 2.22±0.03^c^ |
|  | 1-Penten-3-ol(D) | 0.09±0.01^d^ | 0.08±0.01^de^ | 0.06±0.02^ef^ | 0.04±0^f^ | 0.21±0.01^a^ | 0.17±0.01^c^ | 0.2±0.01^ab^ | 0.19±0^bc^ |
|  | 3-Methyl-3-buten-1-ol | 0.19±0.01^c^ | 0.13±0.02^d^ | 0.09±0.01^e^ | 0.08±0.01^e^ | 0.23±0.01^a^ | 0.2±0.01^bc^ | 0.22±0.01^ab^ | 0.2±0.01^bc^ |
|  | (Z)-2-Penten-1-ol | 0.5±0.02^b^ | 0.38±0.01^c^ | 0.31±0.03^d^ | 0.29±0.02^d^ | 0.6±0.02^a^ | 0.53±0.01^b^ | 0.61±0.02^a^ | 0.58±0.01^a^ |
|  | 1-Hexanol | 0.14±0.01^a^ | 0.1±0.01^b^ | 0.07±0.01^cd^ | 0.07±0^d^ | 0.09±0.01^bc^ | 0.08±0.01^bcd^ | 0.09±0.01^bc^ | 0.09±0.01^bcd^ |
| Total |  | 18.98±0.36^a^ | 14.43±0.86^c^ | 13.77±0.71^c^ | 11.14±0.4^d^ | 15.64±0.21^b^ | 14.46±0.18^c^ | 14.52±0.05^c^ | 13.67±0.04^c^ |
| Esters（2） | Acetic acid ethyl ester | 0.06±0^c^ | 0.04±0.01^d^ | 0.03±0^d^ | 0.02±0^e^ | 0.09±0^a^ | 0.06±0.01^bc^ | 0.08±0.01^a^ | 0.07±0.01^b^ |
|  | Acetic acid propyl ester | 1.3±0.04^a^ | 0.82±0.06^b^ | 0.75±0.02^c^ | 0.57±0.01^d^ | 0.46±0.01^e^ | 0.48±0^e^ | 0.31±0.01^f^ | 0.34±0.01^f^ |
| Total |  | 1.36±0.04^a^ | 0.86±0.06^b^ | 0.78±0.02^c^ | 0.6±0.01^d^ | 0.55±0.01^de^ | 0.54±0.01^e^ | 0.4±0.01^f^ | 0.41±0^f^ |
| Acids（2） | Acetic acid(M) | 6.48±0.43^a^ | 4.55±0.34^b^ | 4.05±0.23^c^ | 3.28±0.15^d^ | 2.95±0.05^de^ | 2.81±0.03^e^ | 2.94±0.07^de^ | 2.72±0.15^e^ |
|  | Acetic acid(D) | 1.33±0.14^a^ | 0.91±0.09^b^ | 0.78±0.06^b^ | 0.58±0.03^c^ | 0.5±0.01^cd^ | 0.49±0.01^cd^ | 0.53±0.04^cd^ | 0.44±0.04^d^ |
| Total |  | 7.81±0.57^a^ | 5.46±0.42^b^ | 4.83±0.29^c^ | 3.86±0.17^d^ | 3.45±0.05^de^ | 3.3±0.04^de^ | 3.46±0.12^de^ | 3.16±0.19^e^ |
| Ethers（2） | Dimethyl sulfide(M) | 14.6±0.35^a^ | 10.38±0.73^b^ | 10.14±0.44^b^ | 8.74±0.22^c^ | 8.65±0.21^c^ | 7.49±0.13^e^ | 8.53±0.07^cd^ | 7.9±0.04^de^ |
|  | Dimethyl sulfide(D) | 1.54±0.09^a^ | 1.66±0.12^a^ | 1.22±0.07^b^ | 1.12±0.03^b^ | 1.12±0.04^b^ | 1.13±0.06^b^ | 0.89±0.02^c^ | 1.22±0.05^b^ |
| Total |  | 16.15±0.44^a^ | 12.04±0.84^a^ | 11.37±0.51^b^ | 9.86±0.24^b^ | 9.78±0.21^b^ | 8.62±0.08^b^ | 9.42±0.05^c^ | 9.11±0.06^b^ |
| Pyridines | 2,6-Dimethylpyridine | 0.13±0.01^a^ | 0.1±0^b^ | 0.09±0.01^b^ | 0.07±0^c^ | 0.1±0.01^c^ | 0.09±0^d^ | 0.1±0.01^cd^ | 0.09±0.01^cd^ |
| Unidentified  （14） | 1 | 0.41±0.06^a^ | 0.46±0.05^a^ | 0.26±0.02^b^ | 0.25±0.04^b^ | 0.12±0^d^ | 0.18±0.01^c^ | 0.11±0^d^ | 0.15±0.01^cd^ |
|  | 2 | 0.17±0.02^b^ | 0.21±0.03^a^ | 0.12±0.02^c^ | 0.15±0.01^bc^ | 0.07±0.01^d^ | 0.07±0^d^ | 0.07±0.01^d^ | 0.07±0^d^ |
|  | 3 | 0.25±0.01^c^ | 0.78±0.05^a^ | 0.15±0.04^d^ | 0.49±0.03^b^ | 0.07±0.01^d^ | 0.08±0.02^d^ | 0.07±0.01^d^ | 0.07±0^d^ |
|  | 4 | 0.88±0.06^a^ | 0.71±0.07^b^ | 0.84±0.02^a^ | 0.57±0.04^c^ | 0.45±0.01^d^ | 0.41±0.01^de^ | 0.35±0.01^e^ | 0.41±0^de^ |
|  | 5 | 0.21±0.04^ab^ | 0.18±0.03^bc^ | 0.25±0.02a | 0.15±0.02^c^ | 0.1±0.01^d^ | 0.09±0.01^d^ | 0.08±0.01^d^ | 0.09±0.01^d^ |
|  | 6 | 0.18±0.03^a^ | 0.13±0.01^c^ | 0.1±0.01^c^ | 0.09±0^b^ | 0.14±0^b^ | 0.13±0.01^b^ | 0.13±0^b^ | 0.13±0^b^ |
|  | 7 | 3.17±0.16^b^ | 3.77±0.23^a^ | 2.16±0.14^c^ | 3±0.11^b^ | 0.56±0.01^d^ | 0.5±0.01^d^ | 0.48±0.02^d^ | 0.49±0.02^d^ |
|  | 8 | 1.72±0.16^c^ | 7.44±0.48^a^ | 1.16±0.1^d^ | 4.96±0.19^b^ | 0.09±0.01^e^ | 0.09±0.01^e^ | 0.1±0.01^e^ | 0.09±0.01^e^ |
|  | 9 | 0.53±0.02^a^ | 0.46±0.03^b^ | 0.4±0.03^c^ | 0.34±0.02^d^ | 0.29±0.01^e^ | 0.38±0.01^c^ | 0.26±0.01^e^ | 0.37±0^cd^ |
|  | 10 | 1.19±0.04^a^ | 0.77±0.04^c^ | 0.93±0.04^b^ | 0.72±0.03^c^ | 0.52±0.01^e^ | 0.56±0.01^de^ | 0.58±0.01^d^ | 0.56±0.01^de^ |
|  | 11 | 0.27±0.02^a^ | 0.22±0.02^b^ | 0.15±0.01^c^ | 0.13±0.01^d^ | 0.1±0^e^ | 0.15±0.01^c^ | 0.06±0^f^ | 0.09±0^e^ |
|  | 12 | 0.4±0.01^a^ | 0.26±0.03^d^ | 0.34±0.01^b^ | 0.2±0.01^e^ | 0.36±0.01^b^ | 0.3±0.02^c^ | 0.35±0.01^b^ | 0.3±0.01^c^ |
|  | 13 | 0.07±0.01^a^ | 0.04±0^de^ | 0.06±0.01^bc^ | 0.03±0^e^ | 0.06±0^b^ | 0.06±0^bc^ | 0.06±0^bc^ | 0.05±0^cd^ |
|  | 14 | 2.8±0.11^a^ | 2.59±0.18^b^ | 1.84±0.08^c^ | 1.66±0.05^de^ | 1.44±0.01^f^ | 1.74±0.02^cd^ | 1.13±0.02^g^ | 1.5±0.03^ef^ |
| Total |  | 12.26±0.51^b^ | 18.02±1.22^a^ | 8.77±0.47^c^ | 12.74±0.55^b^ | 4.38±0.05^d^ | 4.73±0.05^d^ | 3.82±0.08^d^ | 4.37±0.02^d^ |
